# Antimicrobial Effect of Cellulose Nanofibrils (CNFs) and Biobased Additives in Polyvinyl Alcohol Nanocomposite Materials for Sustainable Food Packaging Application

**DOI:** 10.3390/polym18070846

**Published:** 2026-03-31

**Authors:** Fabiola Valdebenito, Carolina Paz Quezada, Danitza Parra, Valentina Rivera, Elizabeth Elgueta, Rodrigo Cáceres, René Cabezas, Carlos Farkas, Miguel Pereira, Laura Azocar, Giovanni Ponce

**Affiliations:** 1 Departamento de Química Ambiental, Facultad de Ciencias, Universidad Católica de la Santísima Concepción, Avenida Alonso de Ribera 2850, Concepción 4090541, Chile; carolina.pqb@gmail.com (C.P.Q.); vrivera@qaciencias.ucsc.cl (V.R.); eelgueta@ucsc.cl (E.E.); rcaceres@ucsc.cl (R.C.); rene.cabezas@ucsc.cl (R.C.); lazocar@ucsc.cl (L.A.); 2 Centro de Energía, Universidad Católica de la Santísima Concepción, Avenida Alonso de Ribera 2850, Concepción 4090541, Chile; 3 Programa de Doctorado en Ciencias con Mención en Biodiversidad y Biorecursos, Facultad de Ciencias, Universidad Católica de la Santísima Concepción, Avenida Alonso de Ribera 2850, Concepción 4090541, Chile; 4 Departamento de Ciencias Básicas y Morfología, Facultad de Medicina, Universidad Católica de la Santísima Concepción, Avenida Alonso de Ribera 2850, Concepción 4090541, Chile; cfarkas@ucsc.cl; 5 Departamento de Ingeniería Química, Universidad de Concepción, Edmundo Larenas 219, Concepción 4030000, Chile; miguelpereira@udec.cl; 6 Unidad de Desarrollo Tecnológico, Universidad de Concepción, Av. Cordillera Nº 3624, Parque Industrial Coronel, Coronel 4191996, Chile; g.ponce@udt.cl

**Keywords:** nanocomposite, PVA/CNFs, antimicrobial properties, packaging food

## Abstract

This study investigates the development of polyvinyl alcohol (PVA)-based nanocomposite films reinforced with cellulose nanofibrils (CNFs) and biobased additives derived from blueberry pruning waste for sustainable food packaging applications. The nanocomposites were fabricated via solvent casting and evaluated in terms of their thermal and antimicrobial properties. Thermogravimetric analysis (TGA/DTG) revealed that the thermal degradation of the nanocomposites occurs through overlapping processes of PVA and CNFs, with maximum degradation temperatures ranging from 273 to 293 °C depending on the formulation. The incorporation of CNFs modified the degradation pathway and promoted the formation of thermally stable carbonaceous residues, while TEMPO-oxidized samples exhibited a decrease in degradation onset (14–24 °C) due to the presence of oxidized surface groups. Remarkably, the nanocomposites exhibited significant antimicrobial activity against both Gram-negative (*Escherichia coli*) and Gram-positive (*Staphylococcus aureus*) bacteria without the incorporation of external antimicrobial agents. Bleached PVA/CNFs films achieved complete growth inhibition (100%), while lignin-containing and additive-modified systems showed selective antibacterial behavior. Zeta potential analysis confirmed a negatively charged CNF surface (−35.3 mV), which may contribute to electrostatic interactions with bacterial membranes. Scanning electron microscopy (SEM) revealed nanostructured surfaces with exposed fibrillar networks that promote bacterial adhesion and immobilization, supporting a contact-active antimicrobial mechanism. These findings demonstrate that the antimicrobial performance of PVA/CNFs nanocomposites is governed by intrinsic physicochemical and topographical properties rather than by the release of antimicrobial agents. This approach provides a safer and more sustainable strategy for the design of active food packaging materials.

## 1. Introduction

Food packaging materials represent a critical component in the preservation, safety, and distribution of food products. In recent years, increasing environmental concerns associated with conventional petroleum-based plastics have driven the development of more sustainable alternatives. In this context, it is important to distinguish between biobased polymers, which are derived from renewable resources, and biodegradable polymers, which are capable of degradation through biological processes, as these terms are often used interchangeably despite representing different concepts [1,2].

Among biodegradable polymers, polyvinyl alcohol (PVA) has attracted significant attention due to its excellent film-forming ability, transparency, mechanical strength, and chemical resistance. Although PVA is synthetically produced, it is biodegradable under specific conditions and has been widely used in food packaging applications [3,4]. However, its high hydrophilicity and limited antimicrobial functionality restrict its direct application in active packaging systems [3]. To overcome these limitations, PVA has been combined with different reinforcing agents and fillers, including inorganic nanoparticles such as silver, zinc oxide, and titanium dioxide, which provide antimicrobial activity but may raise concerns regarding toxicity, migration, and environmental impact [5,6].

In recent years, nanocellulose has emerged as a promising biobased reinforcement material for polymer nanocomposites. Cellulose nanofibrils (CNFs), derived from lignocellulosic biomass, exhibit high aspect ratio, large specific surface area, and excellent mechanical properties, making them suitable for improving the performance of biodegradable polymers [7,8,9]. The incorporation of CNFs into PVA matrices has been shown to enhance mechanical strength, barrier properties, and structural stability through strong hydrogen bonding interactions [10,11,12]. These improvements are mainly attributed to the formation of percolated nanofibrillar networks within the polymer matrix, which restrict polymer chain mobility and reduce gas permeability.

Despite these advantages, the development of antimicrobial nanocellulose-based materials remains a challenging area. Most reported systems rely on the incorporation of external antimicrobial agents, such as metallic nanoparticles, antimicrobial peptides, or bioactive compounds, to achieve effective antibacterial performance [13,14,15]. For instance, nanocellulose-based films loaded with nisin or ethyl lauroyl arginate have demonstrated significant antimicrobial activity against Gram-positive and Gram-negative bacteria; however, these systems depend on the release of active agents, which may lead to reduced long-term effectiveness and potential safety concerns [14,15]. Similarly, metallic nanoparticles such as Ag and ZnO are widely used due to their strong antimicrobial properties, but their application in food packaging is limited by issues related to cytotoxicity, migration, and regulatory constraints [5,13].

In contrast, the intrinsic antimicrobial properties of nanocellulose-based systems without external additives remain relatively underexplored. Recent studies suggest that nanocellulose materials can exhibit antimicrobial behavior through physicochemical interactions with bacterial cells, including surface charge effects, nanoscale roughness, and mechanical interactions at the cell–material interface [16,17]. These mentioned mechanisms are typically described as contact-active antimicrobial behavior, where bacterial inhibition occurs through direct interaction with the material surface rather than through the release of antimicrobial agents.

An additional strategy to enhance the functionality of nanocellulose is the use of unbleached cellulose nanofibrils (L-CNFs), which retain residual lignin. Lignin is a complex aromatic biopolymer known for its antioxidant and antimicrobial properties, mainly due to its phenolic structure [18,19,20]. Recent studies have highlighted lignin as a promising biobased component for active packaging applications, offering improved sustainability and functionality compared to synthetic additives [19]. The presence of lignin in nanocellulose can also influence interfacial interactions, hydrophobicity, and overall composite performance [21].

Based on these considerations, this study proposes the development of PVA/CNFs nanocomposite films reinforced with both bleached and lignin-containing nanofibrils, as well as systems incorporating biobased additives derived from blueberry pruning waste. The central hypothesis is that the combination of nanocellulose surface properties, nanoscale morphology, and lignin-derived functionalities can induce antimicrobial activity without the need for external antimicrobial agents.

**Figure 1:**
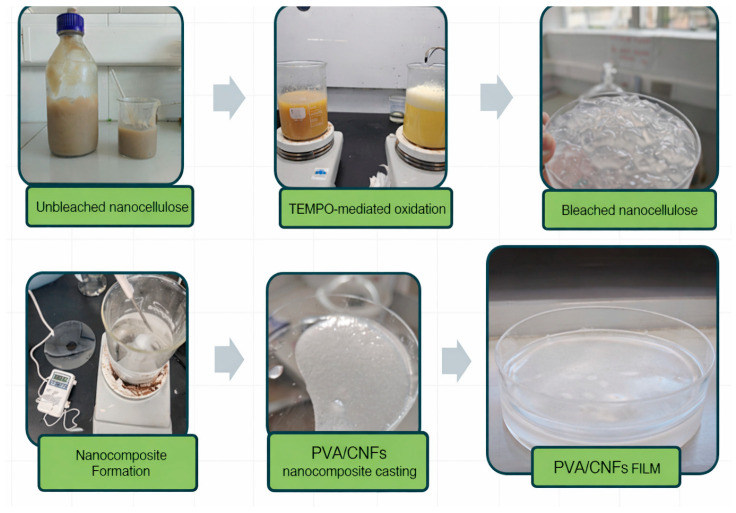
Conceptual diagram of proposed work to develop a green-packaging food with antimicrobial properties.

The objective of this work is therefore to evaluate the thermal behavior and antimicrobial performance of PVA/CNFs nanocomposites, with particular emphasis on understanding the relationship between structure, surface properties, and antibacterial activity. By demonstrating intrinsic antimicrobial behavior in these systems, this study aims to contribute to the development of safer and more sustainable active food packaging materials. A conceptual representation of the proposed approach is presented in Figure 1.

## 2. Materials and Methods

Raw materials and Chemicals

Unbleached nanocellulose hydrogel containing lignin (L-CNFs) was supplied by Bioforest (Celulosa Arauco), Santiago, Chile. The nanocellulose was obtained from unbleached kraft pulp (UKP) derived from *Pinus radiata*, produced at the Arauco pulp mill (Constitución, Chile). This pulp is commonly used as a raw material for a wide range of industrial applications, including filtration materials, packaging products, dielectric papers, fiber-cement materials, and other specialized cellulose-based products.

The UKP originates from sustainably managed *Pinus radiata* plantations. The lignin-containing cellulose nanofibrils (L-CNFs) were produced through a mechanical fibrillation process using an industrial Masuko grinder, resulting in a nanostructured hydrogel with preserved lignin content.

Blueberry pruning wastes were provided by the agricultural company FISTUR (Yumbel, Biobío Region, Chile).

Polyvinyl alcohol (PVA; molecular weight 146,000–186,000 g·mol^−1^; degree of hydrolysis > 99%), 2,2,6,6-tetramethylpiperidine-1-oxyl (TEMPO), NaBr, NaClO, and ethanol were purchased from Merck/Sigma-Aldrich (Santiago, Chile) and used without further purification.

### 2.1. Removable Extraction from Blueberry Pruning (Modified TAPPI T204 Cm-97)

For its determination, 4.0 ± 0.1 g of sample (dry weight) at equilibrium moisture content, prepared according to TAPPI standard 257 cm-85, were used. The raw material was placed in a pre-weighed filter paper cartridge. On top of the cartridge with the sample inside, another cartridge was placed as a lid, also pre-weighed, to prevent sample losses during extraction. The assembly was placed in a Soxhlet, and 150 mL of analytical-grade acetone was added, approximately up to one and a half siphons. The Soxhlet was connected to a flask with a condenser and heated to reflux under a hood, bringing the mixture to a boil. After 6 h, during which no fewer than 24 extractions must occur, and once cooled, the Soxhlet was disassembled, and the solvent in the Erlenmeyer flask was partially evaporated at room temperature under a hood. The cartridge with the sample was collected for subsequent analyses, dried in an oven at 105 °C for 24 h. Finally, it was weighed, and the extracted mass (mf) was obtained [22].

### 2.2. CNFs Obtention

TEMPO-mediated oxidation nanocellulose hydrogels was carried out according to the protocol described by Saíto et al., 2006 [23] with some modifications. A total of 10 g of 3 wt% sample was suspended in 300 mL of deionized water containing 0.050 g TEMPO and 0.50 g NaBr. TEMPO-mediated oxidation began with the addition of 30 mL of a commercial NaClO solution (10 wt%) at room temperature with gentle stirring. The pH was maintained at 10.5 by adding 0.5 M NaOH. The reaction was considered complete once the pH remained stable at 10.5. The oxidized product was filtered, washed with deionized water, and stored at 4 °C [23]. Each experiment was conducted in triplicate. The oxidized fibers were dispersed in deionized water with a pulp concentration of 1 wt.% and then homogenized with a high-pressure homogenizer (GEA PandaPLUS Lab Homogenizer 2000, GEA Mechanical Equipment Italia S.p.A., Parma, Italy) for 8 passes at a pressure drop of 800 bar to produce nanocellulose hydrogels [24]. The resulting CNFs hydrogel obtained after the mechanical treatment is shown in Figure S1 (Supplementary Materials). Films (20 g/m^2^) were prepared in plastic Petri dishes, based on dispersions of 0.25% CNFs. The drying temperature was 24–26 °C [22].

### 2.3. Preparation of PVA/CNFs Nanocomposites

A 5 wt% PVA solution was prepared by dissolving the polymer in distilled water under stirring at 90 °C for 4 h. After cooling to room temperature, a 2 wt% CNFs suspension (bleached, unbleached, or with blueberry extractives) was added and stirred for an additional 4 h. The resulting PVA/CNFs hydrogel is shown in Figure S2 (Supplementary Materials). The final films were cast in Petri dishes and dried under ambient conditions (20–25 °C, 40–60% RH) for approximately 7 days until complete water evaporation; the casting process carried out under laboratory conditions is illustrated in Figure S3 (Supplementary Materials). Each experiment was performed in triplicate. The final CNFs content in the nanocomposites was approximately 28 wt% relative to dry PVA [25]. Table 1 provides the nomenclature of the nanocomposite samples manufactured for this study.

### 2.4. Fourier Transform Infrared Spectroscopy (FTIR)

The IR measurements were performed with an Agilent Tensor 27 instrument (Agilent, Petaling Jaya, Malaysia) in Fourier transform mode (FTIR). The Agilent MicroLab PC software (version IQ/OQ, 21 CFR Part 11 compliant) was used for data acquisition. A total of 40 scans were collected across a spectral range of 400 to 4000 cm^−1^.

### 2.5. Thermo-Gravimetric Analysis (TGA)

Thermal stability and decomposition profiles were determined using a NETZSCH TG 209 F3 Tarsus^®^ thermal analyzer (MB & Cia, Selb, Germany). Approximately 5 mg of each sample was placed in an alumina crucible and heated from room temperature (~25 °C) up to 600 °C under an inert nitrogen atmosphere, at a constant flow rate of 50 mL/min. The heating rate used was 10 °C/min.

### 2.6. Zeta Potential

Zeta potential was performed using zeta-potentiometer (Zetasizer Nano-ZS90, Malvern Instruments, Westborough, MA, USA) at room temperature and a scattering angle of 90°. The Malvern Zetasizer Software version 7.12 was employed to analyze the collected data. A total of 1 mL of the sample was dispersed in deionized water and used as the measurement medium. 

Prior to measurement, the zeta potential cell was carefully filled with the sample, ensuring the absence of air bubbles. External electrodes were cleaned thoroughly to avoid contamination, and the refractive indices of both the solvent (water) and the sample were correctly set in the instrument software. The temperature was maintained at 25 °C throughout the analysis. 

### 2.7. PVA/CNFs Antimicrobial Activity Nanocomposites

**Figure 2:**
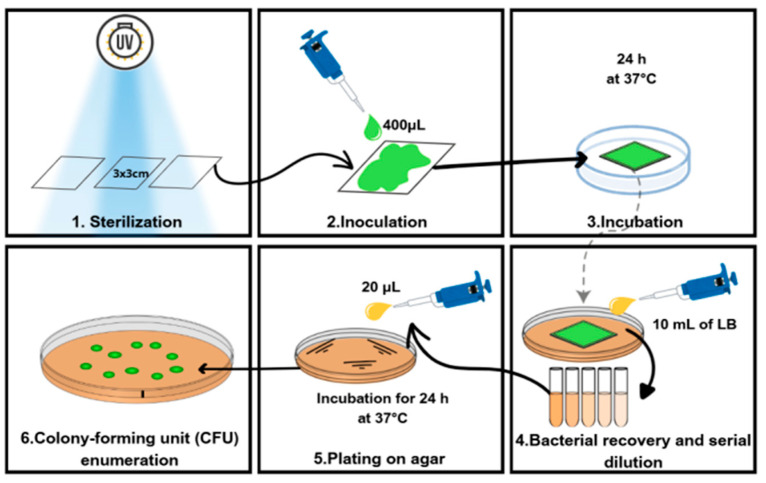
Scheme of the antimicrobial activity JIS test for PVA/CNFs nanocomposites.

The antimicrobial activity of PVA/CNFs nanocomposites was analyzed by modifying the Japanese Industrial Standard (JIS Z 2801:2000) to evaluate antibacterial activity on plastic surfaces. A schematic representation of the antimicrobial assay procedure is shown in Figure 2. The JIS test is based on a comparison of bacterial counts (*S. aureus* and *E. coli*) in saline solution on reference (PVA) and sample materials (PVA + CNFs) after a defined incubation temperature and time (37 °C, 24 h) [26,27]. Materials exhibiting a calculated log10 reduction ≥ 2.0 after 24 h are considered effective antimicrobial agents [26]. Each experiment was performed in triplicate for each nanocomposite.

### 2.8. Scanning Electron Microscopy (SEM) with Energy Dispersive X-Ray Spectroscopy (EDS)

After the bacterial contact period with *S. aureus*, the films were removed and cut into 0.5 cm × 0.5 cm fragments. The samples were transferred to 50 mL Falcon tubes containing 1 mL of 4% paraformaldehyde (PFA) as a fixative agent and incubated for 4 h at room temperature to preserve bacterial morphology and bacteria–surface interactions. Subsequently, the samples were washed three times with phosphate-buffered saline (PBS, pH 7.2) and subjected to gradual dehydration using a graded ethanol series (30, 50, 70, 90, and 100%), with 10 min incubations at each concentration; the absolute ethanol step was repeated. After dehydration, the samples were dried by critical point drying [28].

The dried film fragments were mounted on SEM stubs and coated with a thin gold layer using a sputter coater (SPI-MODULE, West Chester, PA, USA) to ensure surface conductivity. Morphological analysis was evaluated using a scanning electron microscope (JEOL JSM-6380LV, Tokyo, Japan) equipped with an energy-dispersive X-ray spectroscopy (EDS) detector, with image acquisition performed via a computer-connected system.

## 3. Results

### 3.1. Infrared Spectroscopy Analysis (FTIR)

**Figure 3:**
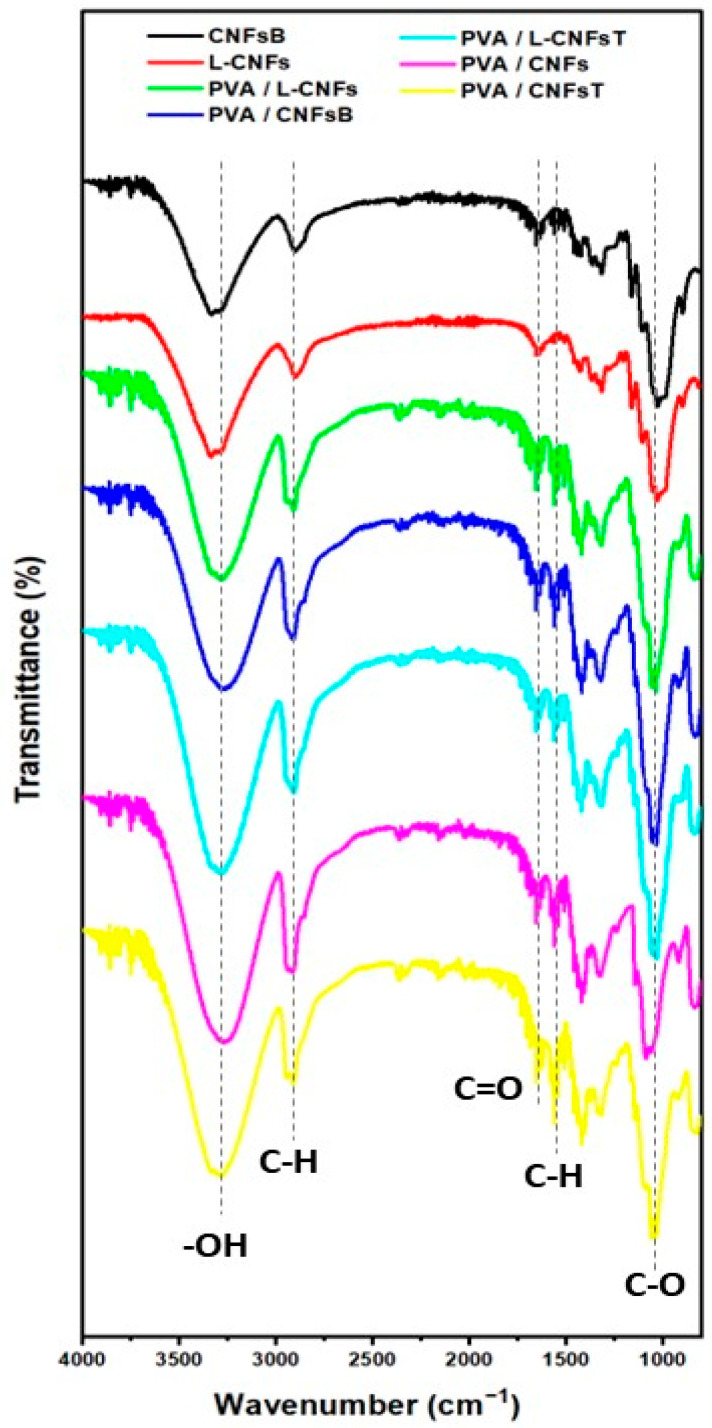
FTIR spectra of PVA/CNFs nanocomposites films.

Figure 3 shows the FTIR spectra of CNFs films and PVA/CNFs nanocomposites. The spectra exhibit the characteristic vibrational bands of both PVA and cellulose nanofibrils, confirming the successful formation of the nanocomposite structure.

A broad absorption band centered around 3300 cm^−1^ was observed in all samples, corresponding to O–H stretching vibrations associated with hydroxyl groups present in both PVA and CNFs. The increased intensity and broadening of this band in the nanocomposites suggest the formation of intermolecular hydrogen bonding interactions between PVA chains and CNFs, which is a well-known mechanism responsible for enhanced compatibility and improved material properties in PVA/nanocellulose systems [8,10,11,12].

The absorption band at 2900 cm^−1^ is attributed to C–H stretching vibrations of aliphatic groups, confirming the presence of both polymer and polysaccharide structures. In addition, the appearance of a band around 1700 cm^−1^ in PVA/CNFs-T and related samples is associated with C=O stretching vibrations, which can be attributed to the introduction of carboxyl groups during TEMPO-mediated oxidation. This modification is consistent with previous studies reporting that TEMPO oxidation selectively converts primary hydroxyl groups of cellulose into carboxyl functionalities, altering the surface chemistry of CNFs [8,9].

The band observed near 1580 cm^−1^ can be associated with C–H bending or asymmetric stretching of carboxylate groups, particularly in oxidized samples, indicating the presence of ionized carboxyl functionalities. This observation further supports the successful chemical modification of CNFs and agrees with reports on oxidized nanocellulose systems [8].

In the fingerprint region, the band around 1100 cm^−1^ corresponds to C–O–C and C–O stretching vibrations typical of cellulose and PVA secondary alcohol groups. The preservation of this band in all nanocomposites indicates that the main polysaccharide backbone structure remains intact after processing.

Overall, the FTIR results confirm that the incorporation of CNFs into the PVA matrix does not introduce new covalent bonds but promotes strong interfacial hydrogen bonding interactions, which play a key role in determining the structural and functional properties of the nanocomposites. Similar spectral features and interaction mechanisms have been widely reported in PVA/nanocellulose systems, where hydrogen bonding dominates the matrix–filler interface and contributes to improved mechanical, barrier, and functional performance [10,11,12].

### 3.2. PVA/CNFs Nanocomposites Thermal Degradation Behavior (TGA)

**Figure 4:**
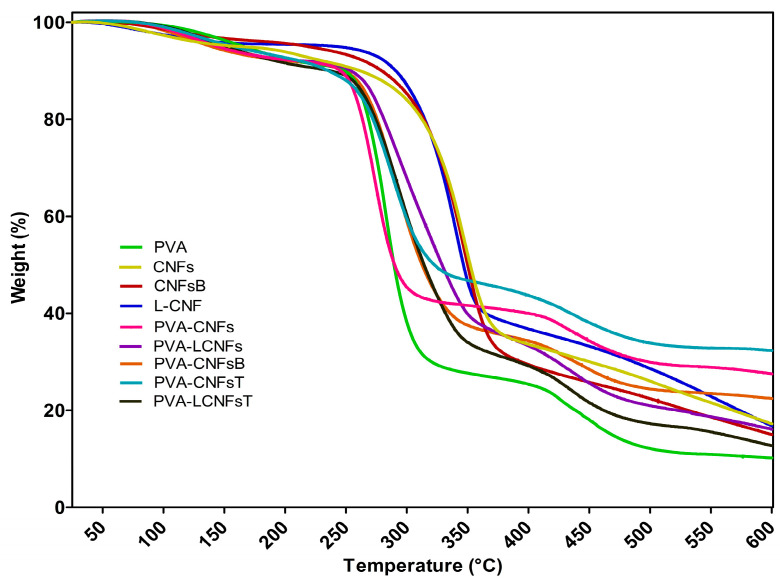

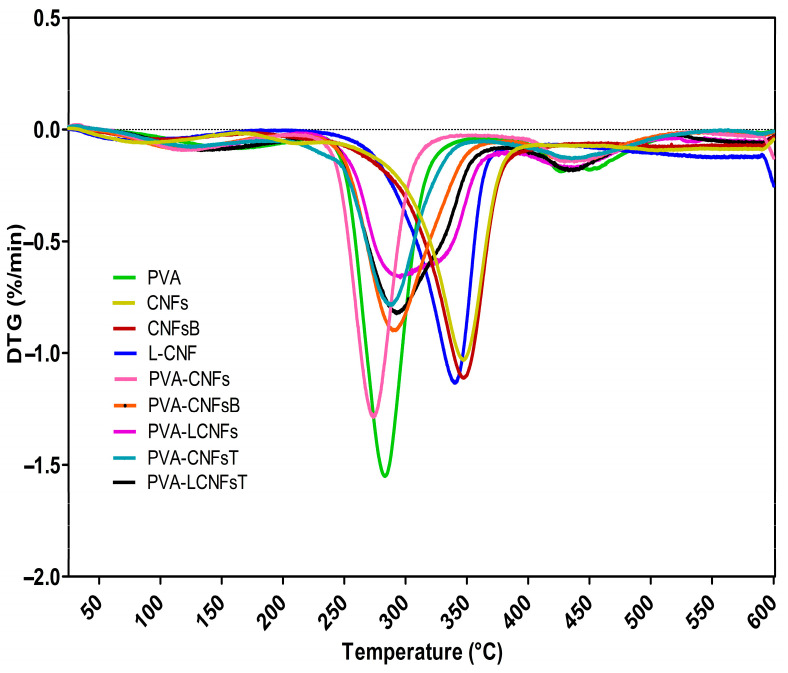
TGA/DTG curves of PVA/CNFs nanocomposite films.

The TGA and DTG analyses (see Figure 4) revealed that the thermal degradation of the films occurs through overlapping processes rather than clearly separated events attributable to each component. Pure PVA exhibited a maximum degradation temperature (Tmax) at 283 °C, confirming its multistep degradation mechanism, which includes dehydration reactions followed by main chain scission.

Neat nanocellulosic materials showed higher thermal stability, with Tmax values of 347 °C (CNFs), 348 °C (CNFsB), and 339 °C (L-CNFs), indicating that cellulose decomposition occurs at higher temperatures than the main degradation step of PVA. However, the onset of degradation (Td10%) for CNFs was observed at 262 °C, confirming that nanocellulose degradation begins well below 350 °C.

For the nanocomposites, the main degradation peak shifted depending on the formulation. The PVA-CNFs sample exhibited the lowest Tmax (273 °C) and Td10% (241 °C), indicating reduced thermal stability. In contrast, PVA-CNFsB (290 °C), PVA-LCNFs (293 °C), PVA-CNFsT (287 °C), and PVA-LCNFsT (293 °C) showed slightly higher Tmax values, suggesting improved thermal resistance depending on CNF type and modification.

These results confirm that the main degradation region between approximately 240 and 350 °C corresponds to overlapping degradation of both PVA and CNFs, rather than independent processes. This behavior is consistent with previous studies reporting that PVA degrades over a broad temperature range while cellulose nanomaterials begin to decompose below 350 °C.

Additionally, the residual mass (char) increased significantly in some nanocomposites, particularly PVA-CNFsT (32%) and PVA-CNFs (27.1%), compared to pure PVA (10%), indicating enhanced formation of thermally stable carbonaceous structures due to the presence of nanocellulose.

TEMPO-oxidized samples showed a lower onset of degradation. Specifically, Td10% decreased from 241 °C (PVA-CNFs) to 227 °C (PVA-CNFsT) and from 241 °C (PVA-LCNFs) to 217 °C (PVA-LCNFsT). This reduction of approximately 14–24 °C can be attributed to the introduction of oxidized surface groups, which promote earlier thermal scission. However, the Tmax values remained relatively similar (287–293 °C), indicating that the main degradation pathway is still governed by the PVA matrix and its interaction with CNFs. A detailed summary of the thermal parameters obtained from TGA analysis is provided in Table S3 (Supplementary Materials).

### 3.3. PVA/CNFs Nanocomposites Zeta Potential (mV) Analysis

In Table 2, it can be observed that CNFs exhibit a strong negatively charged surface, attributed to the presence of hydroxyl and/or carboxylate groups [29], particularly when subjected to TEMPO-mediated oxidation pretreatment. This negative charge of CNFs increases the overall charge of the PVA/CNFs nanocomposite when reinforced with CNFs, considering that the PVA matrix is weakly charged and accounts for approximately 98 wt% of the nanocomposite by mass. Consequently, the charges induced by the small fraction of CNFs in the nanocomposite (2 wt%) can interact with bacterial membranes, particularly those of Gram-positive and Gram-negative bacteria, which differ in their cell membrane structure, contributing to destabilization. Representative zeta potential distributions for the individual components and nanocomposites are provided in Figure S5 (PVA), Figure S6 (CNFs), and Figure S7 (PVA/CNFs nanocomposite) in the Supplementary Materials.

### 3.4. Antimicrobial Activity of PVA/CNFs Nanocomposites

**Figure 5:**
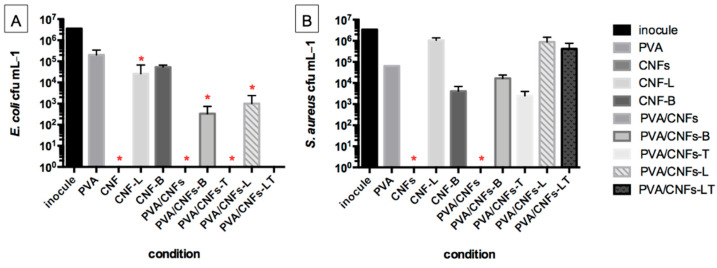
Antibacterial activity against *E. coli* (**A**) and *S. aureus* (**B**) of the PVA/CNFs tested. Those showing a calculated log10 reduction ≥ 2.0 log10 units compared with PVA after 24 h of incubation are marked with a red asterisk.

As shown in Figure 5A all the nanocomposites studied demonstrated an inhibitory effect on Gram-negative bacteria (*E. coli*). Those showing a calculated log10 reduction ≥ 2.0 log10 units compared with PVA after 24 h of incubation are marked with a red asterisk in Figure 5. This means they have a validated antimicrobial activity, according to the JIS 2801 test. The bleached PVA/CNFs tested showed a 100% inhibition of *E. coli* and *S. aureus* growth (Figure 5). This is the same effect observed when using a nanocomposite of CNFs alone. PVA/CNFs-T showed a 100% inhibition of *E. coli* only. The unbleached CNFs (CNF-L), PVA/CNFs-L and PVA/CNFs with blueberries additives tested showed significant antibacterial activity for *E. coli* only. Representative colony count results obtained from the JIS Test are provided in Figure S1 (*S. aureus*) and Figure S2 (*E. coli*) in the Supplementary Materials.

Although CNFs in general do not exhibit antimicrobial properties as strong as those of specific antimicrobial compounds, the nanocomposites with CNFs tested have demonstrated measurable activity, which can be attributed to their topo-chemical and supramolecular characteristics. One key factor is their negatively charged surface, resulting from naturally occurring hydroxyl groups or carboxyl groups introduced during pretreatment. This surface charge can interact with bacterial membranes, leading to membrane disruption. In addition, type II cellulose, which has lower crystallinity (approximately 60%), imparts greater flexibility to the nanofibrils. This flexibility may enhance their ability to interact with bacterial cell walls, contributing to their antimicrobial activity.

### 3.5. Surface Topography and Bacteria–Material Interactions in PVA/CNFs Nanocomposites

**Figure 6:**
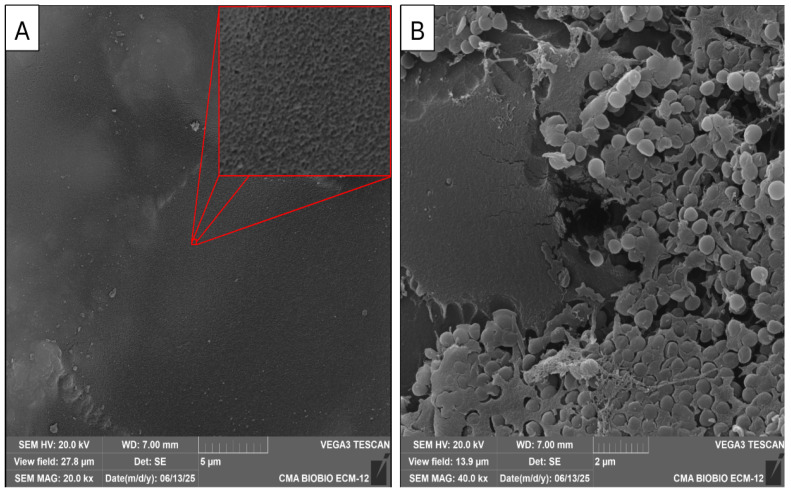
SEM images of PVA/CNFs nanocomposites alone (**A**) or after incubation with *S. aureus* (**B**).

The surface morphology of the PVA/CNFs nanocomposites was examined by scanning electron microscopy (SEM) to elucidate the role of nanoscale topographical features on the antibacterial behaviour of the materials. As shown in Figure 6A, the nanocomposite surface exhibits a heterogeneous and rough morphology, characterized by an interconnected network of cellulose nanofibrils partially exposed at the surface of the PVA matrix. This fibrillar architecture, typical of TEMPO-oxidized and highly fibrillated CNFs, has been widely reported to generate high surface area and pronounced nanoscale roughness [16,17,30,31]. After incubation with *S. aureus* (Figure 6B), bacterial cells are observed adhered and immobilized on the PVA/CNFs surface. The bacteria appear preferentially located in regions with higher nanofibrillar density, indicating that surface topography plays a dominant role in governing bacteria–material interactions. Similar SEM observations have been reported for carboxylated and TEMPO-oxidized CNFs systems, where bacteria are physically entrapped within nanofibrillar networks rather than repelled from the surface [16,30].

The immobilization of *S. aureus* observed in Figure 6B is consistent with a contact-active antibacterial mechanism, previously described for nanocellulose-based materials. In these systems, the antibacterial effect arises from prolonged physical contact between the bacterial cell wall and the nanostructured surface, leading to restricted motility, impaired cell division, and inhibited biofilm formation [30]. SEM images reported [16] showed bacterial cells surrounded and confined by a dense CNFs network, a phenomenon that closely resembles the bacterial immobilization observed in the present PVA/CNFs nanocomposites.

For Gram-positive bacteria such as *S. aureus*, the thick peptidoglycan layer provides mechanical rigidity, which explains the absence of severe cell collapse or lysis in the SEM images. However, this structural robustness also promotes strong physical anchoring to rough and fibrillar surfaces, enhancing immobilization effects. Previous studies have demonstrated that nanocellulose surfaces with high aspect ratio fibrils induce mechanical stress at localized contact points, leading to membrane fatigue and loss of bacterial viability over time, even in the absence of chemical biocides [30].

The topographical contribution to antibacterial activity is further reinforced by studies on oxygenated and highly carboxylated CNFs, where SEM analyses revealed that increased fibrillation degree and surface charge result in denser nanofibrillar networks capable of surrounding bacterial cells and limiting nutrient and oxygen diffusion [16,31]. Although oxygenation was not applied in the present system, the morphological similarities observed by SEM suggest that the PVA/CNFs nanocomposites operate through a comparable physical confinement mechanism.

Importantly, the bacterial adhesion observed in Figure 6B does not indicate a loss of antimicrobial functionality. On the contrary, it supports a non-leaching, contact-based antibacterial mode of action, which is particularly advantageous for food packaging applications. Contact-active antimicrobial surfaces prevent bacterial proliferation directly at the material interface while minimizing the risk of antimicrobial migration into food matrices [14].

Overall, the SEM results confirm that the antibacterial performance of PVA/CNFs nanocomposites is strongly governed by their nanoscale surface topography. The combination of nanofibrillar architecture, surface roughness, and physical confinement of bacterial cells aligns well with previously reported antibacterial mechanisms of TEMPO-oxidized, carboxylated, and oxygenated CNFs [16,17,30,31]. These findings highlight the potential of PVA/CNFs nanocomposites as contact-active antimicrobial materials for sustainable food packaging applications.

## 4. Discussion

The results obtained in this study demonstrate that the performance of PVA/CNFs nanocomposites is not governed by a single dominant factor, but rather by a multiscale structure–function relationship, where thermal behavior, surface chemistry, and nanoscale morphology act synergistically to define both stability and antimicrobial performance.

From a thermal perspective, the TGA/DTG analysis confirmed that the degradation of PVA/CNF systems occurs through overlapping processes rather than discrete events attributable exclusively to each component. Pure PVA exhibited a Tmax at approximately 283 °C, consistent with its multistep degradation mechanism involving dehydration and backbone scission reactions [1,3]. In contrast, nanocellulosic materials showed higher Tmax values (339–348 °C), although their degradation onset occurred below 300 °C, confirming that cellulose decomposition begins well below 350 °C [2,3,9].

In the nanocomposites, the main degradation region between 240 and 350 °C corresponds to the overlapping degradation of both PVA and CNFs, in agreement with previous reports on PVA/nanocellulose systems [2,4,9]. The incorporation of CNFs led to formulation-dependent behavior, where some systems showed reduced Tmax (e.g., PVA/CNFs), while others exhibited slight improvements depending on CNF chemistry and interfacial interactions. This variability highlights that thermal stability is not solely dictated by the filler itself, but by the matrix–filler interaction and dispersion state.

In addition, the presence of lignin and phenolic extractives appears to contribute to enhanced thermal resistance, likely due to their radical scavenging capacity and aromatic structure, which can delay thermal degradation processes. Similar effects have been reported in lignin-containing systems, where phenolic functionalities improve thermal stability and oxidative resistance [10,11,12]. This aspect is particularly relevant for food packaging applications, where thermal stability during processing is a critical requirement.

From a structural perspective, FTIR analysis confirmed the presence of strong hydrogen bonding interactions between PVA and CNFs, as evidenced by the broad O–H stretching band. These interactions play a key role in improving interfacial compatibility and cohesion within the polymer matrix, leading to more homogeneous nanocomposite structures [2,4]. Importantly, these intermolecular interactions not only influence mechanical and thermal properties but also define the surface characteristics that ultimately govern bacteria–material interactions.

At the colloidal level, zeta potential measurements indicated that CNFs possess a highly negative surface charge (−35.3 mV), which is partially transferred to the nanocomposite system. Although the overall surface charge of the PVA matrix remains moderate, the presence of localized negatively charged domains can significantly influence bacterial adhesion and interaction. Electrostatic interactions between nanocellulose surfaces and bacterial membranes have been widely reported as a key factor in antimicrobial performance, particularly in systems with heterogeneous charge distribution [9,10].

However, the most critical factor governing the antibacterial behavior observed in this study is the surface topography at the nanoscale, as revealed by SEM analysis. The formation of a fibrillar, highly rough surface due to the partial exposure of CNFs creates a high-surface area interface with pronounced nanoscale features. Such topographies have been shown to induce mechanical stress, membrane deformation, and restricted bacterial mobility [16,17].

In this context, the antimicrobial activity of the PVA/CNFs nanocomposites can be described as a contact-active mechanism, where bacterial inhibition occurs through direct interaction with the nanostructured surface rather than through the release of antimicrobial agents. SEM observations provide direct evidence of this mechanism, showing bacterial cells adhered and immobilized within the nanofibrillar network. This behavior has been associated with reduced cell division, inhibition of biofilm formation, and eventual loss of viability due to mechanical fatigue and limited nutrient diffusion.

This mechanism differs fundamentally from conventional antimicrobial packaging systems, which typically rely on the incorporation and release of active agents such as metallic nanoparticles, essential oils, or antimicrobial peptides [5,13]. While these systems can be highly effective, they often suffer from limitations such as reduced long-term efficiency, potential toxicity, and regulatory concerns related to migration into food matrices [14,15].

In contrast, the nanocomposites developed in this study operate through a non-leaching antimicrobial mechanism, which offers significant advantages in terms of safety and durability. By eliminating the release step, these materials provide more stable long-term performance while minimizing the risk of contamination [18,19]. This approach aligns with emerging trends in sustainable material design, where functionality is derived from intrinsic physicochemical properties rather than external additives.

The observed differences in antibacterial response between *E. coli* and *S. aureus* further support this mechanism. Gram-negative bacteria, characterized by thinner and more flexible cell envelopes, are more susceptible to mechanical and physicochemical stress induced by nanostructured surfaces, whereas Gram-positive bacteria exhibit greater resistance due to their thicker cell walls [20,21].

Overall, this study establishes a clear structure–function–activity relationship in PVA/CNFs nanocomposites, demonstrating that nanoscale surface engineering is a key strategy for developing next-generation antimicrobial materials. The findings highlight the potential of designing intrinsically active, non-leaching systems for sustainable food packaging applications, reducing reliance on conventional biocidal additives while maintaining high performance.

## 5. Conclusions

PVA/CNFs nanocomposites were successfully developed as biobased antimicrobial materials, and their antibacterial performance was shown to be strongly governed by nanoscale surface topography rather than by the release of antimicrobial agents. The incorporation of cellulose nanofibrils into the PVA matrix generated heterogeneous and rough nanostructured surfaces with exposed nanofibrillar networks, which played a decisive role in bacteria–material interactions.

SEM analysis provided direct evidence that *Staphylococcus aureus* cells adhered and become immobilized on the surface of the PVA/CNFs nanocomposites. This immobilization is attributed to a contact-active antibacterial mechanism, where nanoscale roughness and fibrillar architecture restrict bacterial mobility and proliferation at the interface. These findings are consistent with previously reported antibacterial behaviors of TEMPO-oxidized, carboxylated, and oxygenated cellulose nanofibrils, in which physical confinement within nanofibrillar networks is a dominant mode of action.

Importantly, the observed bacterial adhesion does not indicate a loss of antimicrobial efficacy but rather confirms a non-leaching and physically driven antibacterial mechanism. This characteristic is particularly advantageous for food packaging applications, as it minimizes the risk of antimicrobial migration into food products while ensuring localized and sustained inhibition of bacterial growth upon contact.

Overall, this study demonstrates that controlling nanoscale surface topography is a key strategy for designing effective antimicrobial polymer nanocomposites based on cellulose nanofibrils. The results highlight the potential of PVA/CNFs nanocomposites as sustainable, contact-active antimicrobial materials for advanced food packaging systems, contributing to improved food safety and extended shelf life.

## Figures and Tables

**Table 1:** Nanocomposite nomenclature.

Code	Composition	Description
CNFs	Cellulose nanofibrils	Film prepared from bleached cellulose nanofibrils
CNFs-L	Lignin-CNFs	Film prepared from lignin-containing nanofibrils
CNFs-B	CNFs + blueberry extract	CNFs film with biobased additive from blueberry pruning waste
PVA	Polyvinyl alcohol	Pure PVA film
PVA/CNFs	PVA + CNFs	Nanocomposite film reinforced with cellulose nanofibrils
PVA/CNFs-L	PVA + lignin- CNFs	Nanocomposite reinforced with L-CNFs
PVA/CNFs-T	PVA + TEMPO-CNFs	Nanocomposite with oxidized nanofibrils
PVA/CNFs-LT	PVA + lignin-CNFs (TEMPO)	Nanocomposite with lignin-TEMPO-oxidized CNFs
PVA/CNFs-B	PVA + CNFs + blueberry extract	Nanocomposite with biobased additive from pruning waste

**Table 2:** Zeta potential (mV) of PVA, CNFs and PVA/CNFs samples.

Sample	Zeta Potential (mV)
PVA	−1.307
CNFs-T	−35.29
PVA/CNFs-T	−3.766

## Data Availability

The original contributions presented in this study are included in the article. Further inquiries can be directed at the corresponding author.

## Supplementary Materials

The following supporting information can be downloaded at: https://www.mdpi.com/article/10.3390/polym18070846/s1, Figure S1: CNFs hydrogel; Figure S2: PVA/CNFs hydrogel; Figure S3: PVA/CNFs casting process; Table S1: *S. aureus* Colony count using JIS test; Figure S4: Colony count using JIS test; Table S2: *E. coli* Colony count using JIS test; Figure S5: Zeta Potential of PVA hydrogel sample (5 wt%); Figure S6: Zeta Potential of CNFs hydrogel sample (2 wt%); Figure S7: Zeta Potential of PVA/CNFs hydrogel nanocomposite sample, Table S3. TGA nanocomposite PVA/CNFs analysis data.
