# Supplementary material for: Antimicrobial Effect of Cellulose Nanofibrils (CNFs) and Biobased Additives in Polyvinyl Alcohol Nanocomposite Materials for Sustainable Food Packaging Application
Source: Polymers (Basel). 2026 Mar 31;18(7):846. doi: 10.3390/polym18070846 (PMC13075142; doi:10.3390/polym18070846)
Supplement: Supplementary file 1 [file polymers-18-00846-s001.zip › polymers-4209480-supplementary.pdf]

# Antimicrobial effect of cellulose nanofibrils (CNFs) and bio-based additives in polyvinyl alcohol nanocomposite materials for sustainable food packaging application

**Fabiola Valdebenito [1,2,*], Carolina Paz Quezada [1], Danitza Parra [1], Valentina Rivera [1,3], Elizabeth Elgueta [1,2], Rodrigo Cáceres [1], René Cabezas [1,2], Carlos Farkas [4], Miguel Pereira [5], Laura Azocar [1,2] and Giovanni Ponce [6]**

[1] Departamento de Química Ambiental, Facultad de Ciencias, Universidad Católica de la Santísima
Concepción, Avenida Alonso de Ribera 2850, Concepción, 4090541, Chile; carolina.pqb@gmail.com (C.P-Q.); danitza.pgarcia@gmail.com (D.P.); vrivera@qaciencias.ucsc.cl (VR.); eelgueta@ucsc.cl (E.E.); rcaceres@ucsc.cl (R.O.C.); rene.cabezas@ucsc.cl (R.C.); lazocar@ucsc.cl (L.A.)

[2] Centro de Energía, Universidad Católica de la Santísima Concepción, , Concepción, Avenida Alonso de Ribera 2850, 4090541, Chile; eelgueta@ucsc.cl (E.E); rene.cabezas@ucsc.cl (R.C.); lzocar@ucsc.cl (L.A.)

[3] Programa de Doctorado en Ciencias con Mención en Biodiversidad y Biorecursos, Facultad de Ciencias, Universidad Católica de la Santísima Concepción, Avenida Alonso de Ribera 2850, Concepción, 4090541, Chile; vrivera@qacie cias.ucsc.cl (VR.)

[4] Departamento de Ciencias Básicas y Morfología, Facultad de Medicina, Universidad Católica de la Santísima Concepción, Avenida Alonso de Ribera 2850, Concepción, 4090541, Chile; cfarkas@ucsc.cl (C.F)

[5] Departamento de Ingeniería Química, Universidad de Concepción, Edmundo Larenas 219, Concepción 4030000, Chile; miguelpereira@udec.cl (M.P)

[6] Unidad de Desarrollo Tecnológico, Universidad de Concepción, Av. Cordillera Nº 3624 - Parque Industrial Coronel, Coronel, 4191996, Chile; g.ponce@udt.cl (G.P)

* Correspondence: fvaldebenito@ucsc.cl (F.V); Tel.: +56-412347150

# Supporting Information

## Content:

| | |
|---|---|
| Pages | 7 |
| Figures | 7 |
| Tables | 3 |

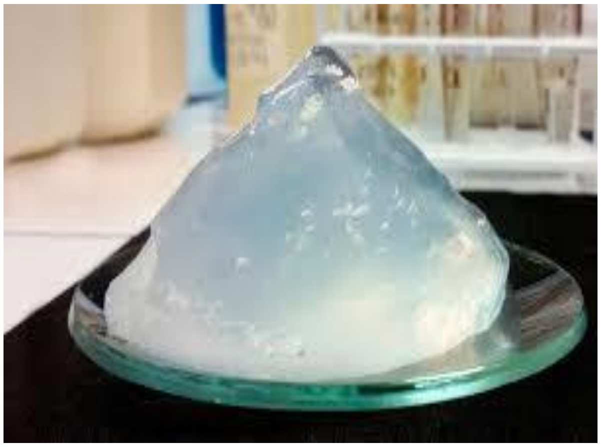

Figure S1. CNFs  hydrogel

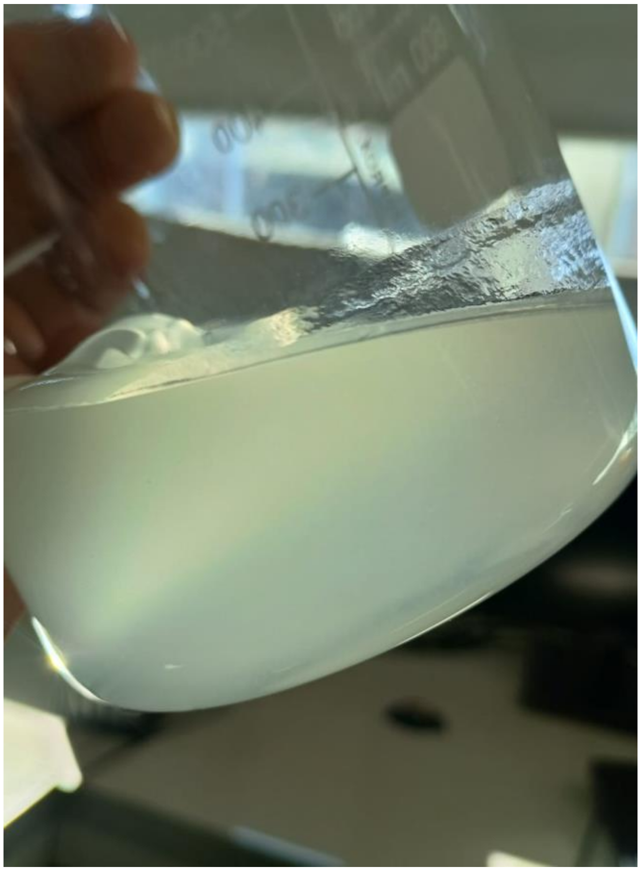

Figure S2. PVA/CNFs hydrogel

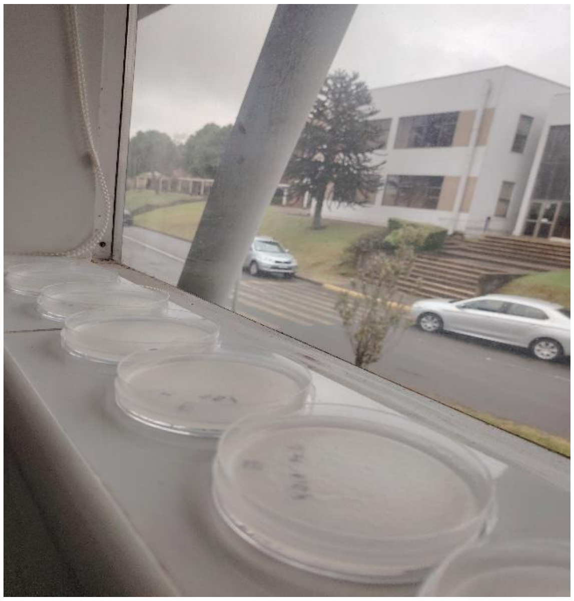

Figure S3. PVA/CNFs casting process

## Table S1. *S. aureus* Colony count using JIS test

| 33 | S. aureus UFC/mL iniciales = | 8,50E+07 | | UFC contadas | | | UFC/mL | promedio | SD | log10(ufc) | log10 -red | | | sinonimo |
|---|---|---|---|---|---|---|---|---|---|---|---|---|---|---|
| 34 | en los 10 mL | 3,40E+06 | -1 | -2 | -3 | -4 | | | | 6,5 | | | | |
| 35 | Lignin-CNFs film | CNF-L | NC | NC | 85 | 47 | 850000 | 1,05E+06 | 3,27E+05 | 5,9 | -1,1 | 6,0 | -1,2 | |
| 36 | | | NC | NC | 143 | 66 | 1430000 | | | 6,2 | -1,3 | | | |
| 37 | | | NC | NC | 88 | 34 | 880000 | | | 5,9 | -1,1 | | | |
| 38 | CNFs film additivity with blueberry pruning removab | CNF-B | 24 | 3 | 2 | 3 | 2400 | 4,10E+03 | 2,77E+03 | 3,4 | 1,4 | 3,6 | 1,3 | CNFs-P |
| 39 | | | 73 | 20 | 7 | 0 | 7300 | | | 3,9 | 0,9 | | | |
| 40 | | | 26 | 7 | 0 | 0 | 2600 | | | 3,4 | 1,4 | | | |
| 41 | PVA/CNFs nanocomposite | PVA/CNFs | 17 | 0 | 0 | 0 | 0 | 0,00E+00 | 0,00E+00 | | **4,8** | | 4,8 | PVA/CNFs- B |
| 42 | | | 0 | 0 | 0 | 0 | | | | | **4,8** | | | |
| 43 | | | 0 | 0 | 0 | 0 | | | | | **4,8** | | | |
| 44 | PVA/CNFs additivity with blueberry pruning removal | PVA/CNFs-B | 129 | 95 | 63 | 41 | 12900 | 1,66E+04 | 7,29E+03 | 4,1 | 0,7 | 4,2 | 0,6 | PVA/CNFs-P |
| 45 | | | 119 | 27 | 0 | 1 | 11900 | | | 4,1 | 0,7 | | | |
| 46 | | | 250 | 141 | 32 | 0 | 25000 | | | 4,4 | 0,4 | | | |
| 47 | PVA/TEMPO-CNFs nanocomposite | PVA/CNFs-T | 13 | 3 | 0 | 0 | 1300 | 2,47E+03 | 1,53E+03 | 3,1 | 1,7 | 3,3 | 1,5 | PVA/CNFs-B (TEMPO) |
| 48 | | | 42 | 13 | 0 | 0 | 4200 | | | 3,6 | 1,2 | | | |
| 49 | | | 19 | 0 | 0 | 0 | 1900 | | | 3,3 | 1,5 | | | |
| 50 | PVA/lignin-CNFs nanocomposite | PVA/CNFs-L | NC | NC | 150 | 58 | 1500000 | 8,77E+05 | 6,01E+05 | 6,2 | -1,4 | 5,9 | -1,1 | PVA/CNFs-L |
| 51 | | | NC | 52 | 30 | 3 | 300000 | | | 5,5 | -0,7 | | | |
| 52 | | | NC | 78 | 83 | 21 | 830000 | | | 5,9 | -1,1 | | | |
| 53 | PVA/lignin-CNFs-TEMPO nanocomposite | PVA/CNFs-LT | 170 | 50 | 7 | 1 | 70000 | 4,20E+05 | 3,40E+05 | 4,8 | 0,0 | 5,5 | -0,6 | PVA/CNFs-L (TEMPO) |
| 54 | | | NC | NC | 75 | 32 | 750000 | | | 5,9 | -1,1 | | | |
| 55 | | | 122 | 57 | 44 | 14 | 440000 | | | 5,6 | -0,8 | | | |
| 56 | PVA | | | 32 | | | 64000 | 64000 | | 4,8 | | | | |
| 57 | | | | 32 | | | 64000 | | | | | | | |

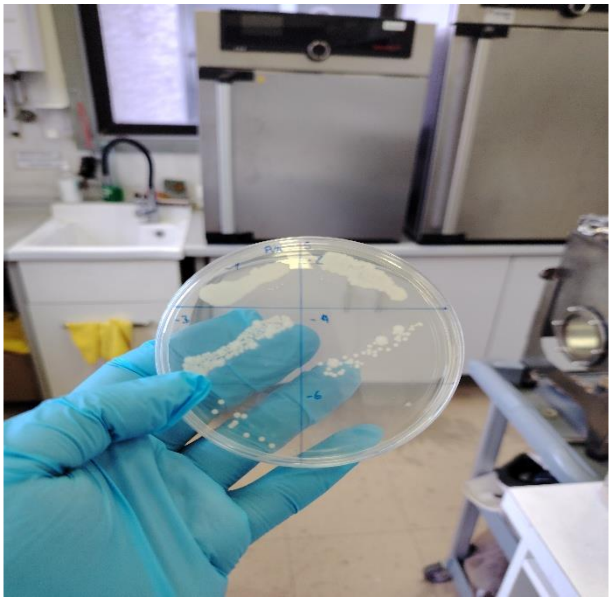

Figure SI-4. Colony count using JIS test

## Table S2. *E. Coli* Colony count using JIS test

| A | B | C | D | E | F | G | H | I | J | K | L | M | N | O |
|---|---|---|---|---|---|---|---|---|---|---|---|---|---|---|
| **E. coli UFC/mL iniciales =** | 9,00E+07 | | **UFC contadas** | | | **UFC/mL** | **promedio** | **SD** | log10(ufc) | log10 -red | **cálculo=** | (1000*n*F | sinonimo | |
| en los 10 mL | 3,60E+06 | **-1** | **-2** | **-3** | **-4** | | | | **6,6** | | | | | |
| Lignin-CNFs film | CNF-L | NC | 73 | 15 | | 73000 | 2,60E+04 | 4,08E+04 | 4,9 | 0,0 | 2,9 | **2,0** | | |
| | | 24 | 5 | 0 | | 5000 | | | 3,7 | 1,1 | | | | |
| | | 4 | 0 | 0 | | 0 | | | 0,0 | 4,8 | | | | |
| CNFs film additivity with blueberry pruning removab | CNF-B | NC | 38 | 8 | | 38000 | 5,33E+04 | 1,33E+04 | 4,6 | 0,2 | 4,7 | 0,1 | CNFs-P | |
| | | NC | 60 | 11 | | 60000 | | | 4,8 | 0,0 | | | | |
| | | NC | 62 | 15 | | 62000 | | | 4,8 | 0,0 | | | | |
| PVA/CNFs nanocomposite | PVA/CNFs | 0 | 0 | 0 | | 0 | 0,00E+00 | 0,00E+00 | 0 | 4,8 | 0,0 | **4,8** | PVA/CNFs- B | |
| | | 0 | 0 | 0 | | 0 | | | 0 | 4,8 | | | | |
| | | 0 | 0 | 0 | | 0 | | | 0 | 4,8 | | | | |
| PVA/CNFs additivity with blueberry pruning removab | PVA/CNFs-B | 1 | 0 | 0 | | 100 | 3,33E+02 | 4,04E+02 | 2 | 2,8 | 2,3 | **2,3** | PVA/CNFs-P | |
| | | 8 | 2 | 0 | | 800 | | | 2,9 | 1,9 | | | | |
| | | 1 | 0 | 0 | | 100 | | | 2 | 2,8 | | | | |
| PVA/TEMPO-CNFs nanocomposite | PVA/CNFs-T | 0 | 0 | 0 | | 0 | 0,00E+00 | 0,00E+00 | 0 | 4,8 | 0,0 | **4,8** | PVA/CNFs-B (TEMPO) | |
| | | 0 | 0 | 0 | | 0 | | | 0 | 4,8 | | | | |
| | | 0 | 0 | 0 | | 0 | | | 0 | 4,8 | | | | |
| PVA/lignin-CNFs nanocomposite | PVA/CNFs-L | 26 | 0 | 0 | | 2600 | 1,00E+03 | 1,39E+03 | 3,4 | 1,4 | 2,7 | **2,1** | PVA/CNFs-L | |
| | | 2 | 0 | 0 | | 200 | | | 2,3 | 2,5 | | | | |
| | | 2 | 1 | 0 | | 200 | | | 2,3 | 2,5 | | | | |
| PVA/lignin-CNFs-TEMPO nanocomposite | PVA/CNFs-LT | 0 | 0 | 0 | | 0 | 0,00E+00 | 0,00E+00 | 0 | 4,8 | 0,0 | **4,8** | PVA/CNFs-L (TEMPO) | |
| | | 0 | 0 | 0 | | 0 | | | 0 | 4,8 | | | | |
| | | 0 | 0 | 0 | | 0 | | | 0 | 4,8 | | | | |
| PVA | | 47 | 15 | 2 | | 30000 | 6,60E+04 | | | | | | | |
| E. coli | | | 51 | | | 102000 | | | | | | | | |

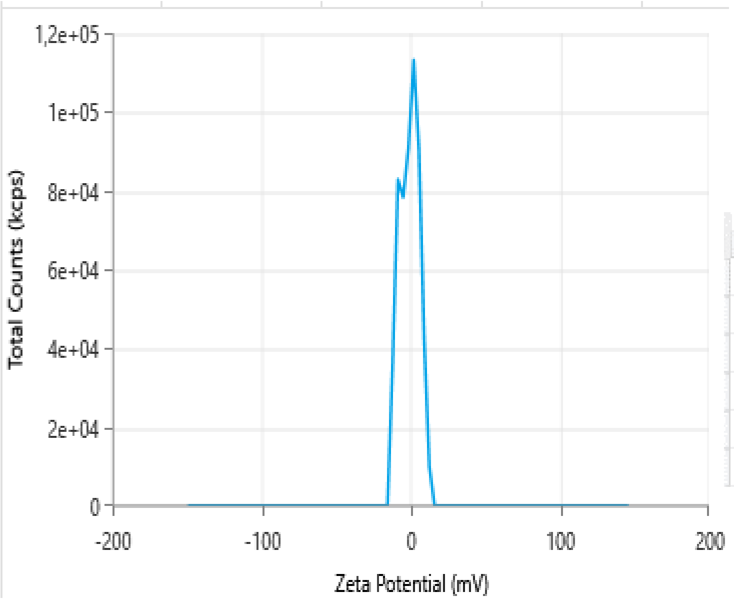


| Name | Mean | Standard Deviation | RSD | Minimum | Maximum |
|---|---|---|---|---|---|
| Zeta Potential (mV) | -1,307 | - | - | -1,307 | -1,307 |
| Conductivity (mS/cm) | 0,9191 | - | - | 0,9191 | 0,9191 |
| Wall Zeta Potential (mV) | 0,1652 | - | - | 0,1652 | 0,1652 |
| Quality Factor | 0,4392 | - | - | 0,4392 | 0,4392 |
| Zeta Peak 1 Mean (mV) | -8,297 | - | - | -8,297 | -8,297 |
| Zeta Peak 2 Mean (mV) | 1,172 | - | - | 1,172 | 1,172 |

Figure S5. Zeta Potential of PVA hydrogel sample (5% wt.)

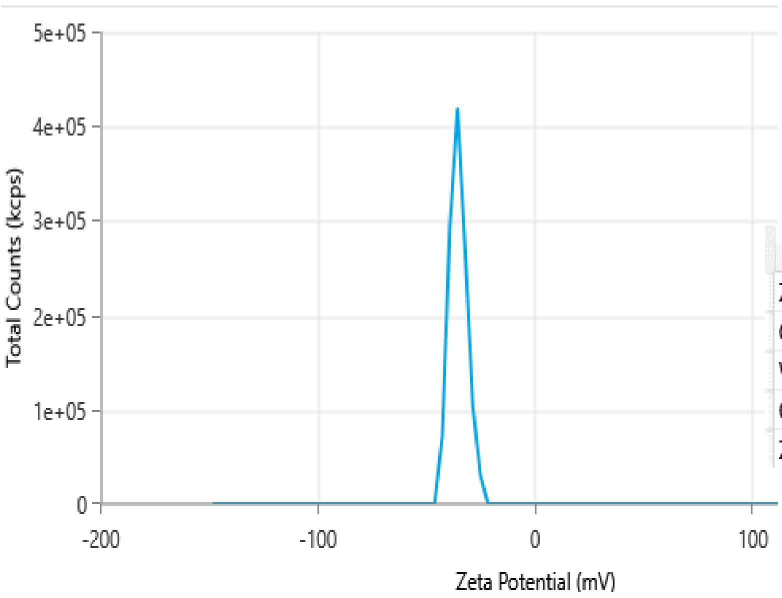


| Name | Mean | Standard Deviation | RSD | Minimum | Maximum |
|---|---|---|---|---|---|
| Zeta Potential (mV) | -35,29 | - | - | -35,29 | -35,29 |
| Conductivity (mS/cm) | 0,02681 | - | - | 0,02681 | 0,02681 |
| Wall Zeta Potential (mV) | 2,458 | - | - | 2,458 | 2,458 |
| Quality Factor | 13,71 | - | - | 13,71 | 13,71 |
| Zeta Peak 1 Mean (mV) | -35,29 | - | - | -35,29 | -35,29 |

Figure S6. Zeta Potential of CNFs hydrogel sample (2% wt.)

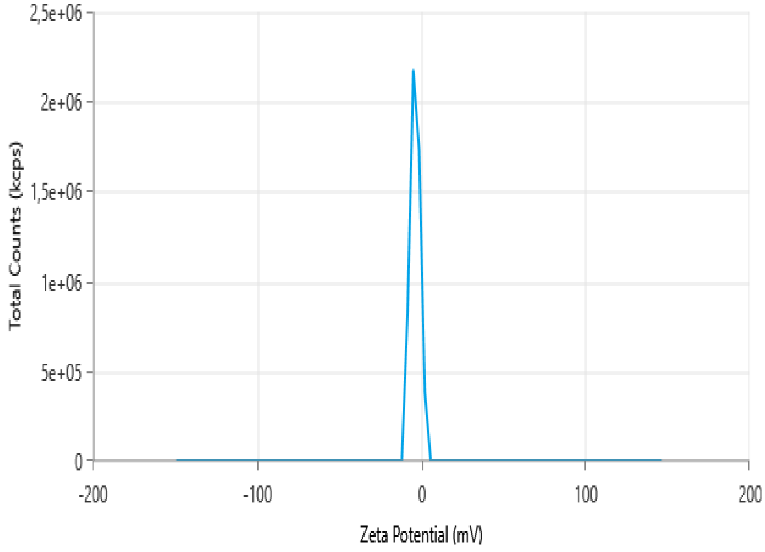


| Name | Mean | Standard Deviation | RSD | Minimum | Maximum |
|---|---|---|---|---|---|
| Zeta Potential (mV) | -3,766 | - | - | -3,766 | -3,766 |
| Conductivity (mS/cm) | 0,5555 | - | - | 0,5555 | 0,5555 |
| Wall Zeta Potential (mV) | 1,474 | - | - | 1,474 | 1,474 |
| Quality Factor | 1,013 | - | - | 1,013 | 1,013 |
| Zeta Peak 1 Mean (mV) | -3,766 | - | - | -3,766 | -3,766 |

Figure S7. Zeta Potential of PVA/CNFs hydrogel nanocomposite sample.

## **Table S3.** *TGA nanocomposite PVA/CNFs analysis data*

| Sample | Tmáx (°C) | $T_{d10\%}$ (°C) | char (%) |
|---|---|---|---|
| PVA | 283 | 288 | 10,0 |
| CNFs | 347 | 262 | 17,0 |
| CNFsB | 348 | 276 | 14,6 |
| L-CNFs | 339 | 288 | 16,4 |
| PVA-CNFs | 273 | 241 | 27,1 |
| PVA-CNFsB | 290 | 240 | 22,5 |
| PVA-LCNFs | 293 | 241 | 15,8 |
| PVA-CNFsT | 287 | 227 | 32,0 |
| PVA-LCNFsT | 293 | 217 | 12,7 |
